# Keratinocyte stimulation of matrix metalloproteinase-1 production and proliferation in fibroblasts: regulation through mitogen-activated protein kinase signalling events

**DOI:** 10.1038/sj.bjc.6600478

**Published:** 2002-08-12

**Authors:** S E Moon, N Bhagavathula, J Varani

**Affiliations:** Department of Pathology, The University of Michigan Medical School, 1301 Catherine Road, Box 0602, Ann Arbor, Michigan, MI 48109, USA

## Abstract

Incubation of human dermal fibroblasts in keratinocyte-conditioned culture medium led to a 5.7-fold increase in the level of matrix metalloproteinase-1. Virtually all of the matrix metalloproteinase-1 – inducing activity could be related to agonists acting through members of the epidermal growth factor receptor family or to agonists acting through the interleukin-1 receptor. The same keratinocyte-conditioned medium also induced a modest increase in fibroblast proliferation (approximately 1.8-fold). Growth-stimulating activity could be attributed to epidermal growth factor receptor (but not interleukin-1 receptor) function. In fibroblasts exposed to keratinocyte-conditioned medium, mitogen-activated protein kinase signalling through both the extracellular signal-related kinase pathway and p38 pathway occurred. When recombinant epidermal growth factor or recombinant interleukin-1β were used as a control, they induced mitogen-activated protein kinase signalling consistent with the combined effects of epidermal growth factor receptor – specific and interleukin-1 receptor – specific agonists in keratinocyte-conditioned medium. Recombinant epidermal growth factor stimulated both matrix metalloproteinase-1 induction and proliferation while recombinant interleukin-1β stimulated matrix metalloproteinase-1 elaboration but not fibroblast growth. An inhibitor of extracellular signal-related kinase pathway signalling (U0126) blocked induction of matrix metalloproteinase-1 production induced by keratinocyte-conditioned medium (as well as by epidermal growth factor or interleukin-1β), and also inhibited proliferation. A p38 signalling inhibitor (SB203580) blocked matrix metalloproteinase-1 elaboration induced by keratinocyte-conditioned medium or interleukin-1β, but did not inhibit matrix metalloproteinase-1 elaboration or cell growth induced by epidermal growth factor. These data indicate that keratinocyte-fibroblast interactions are mediated by multiple stimulating agents acting on specific receptors to induce signalling through different mitogen-activated protein kinase pathways leading to altered expression of key biological functions.

*British Journal of Cancer* (2002) **87**, 457–464. doi:10.1038/sj.bjc.6600478 www.bjcancer.com

© 2002 Cancer Research UK

## 

Epithelial tumour cell penetration into the stroma during invasion requires degradation of the basement membrane and the underlying structural collagen. Matrix metalloproteinases (MMPs) with collagenolytic activity are thought to be responsible for much of the connective tissue damage that occurs during invasion (Bauer *et al*, 1977; Goslen and Bauer 1986; Muller *et al*, 1993; Gray *et al*, 1992; Varani *et al*, 2000). The tumour cells, themselves, are a source of MMPs (Wright *et al*, 1994; Putnins *et al*, 1996), but a variety of evidences suggests that cells of the stromal tissue surrounding the tumour comprise a major source of tissue-destructive MMPs (Kataoka *et al*, 1993; Heppner *et al*, 1996; Declerck, 2000).

We have utilised a human skin organ culture model to study the process of stromal invasion by epithelial cells and the role of MMPs in this process. In this invasion model, human skin is maintained in organ culture for a period of 8–12 days under serum-free, growth factor-free conditions or in the presence of exogenous growth-promoting factors. When the organ culture medium contains no serum or exogenous growth factor, normal histological structure and biochemical function are maintained (Varani *et al*, 1993, 1994). However, when epidermal growth factor (EGF) is included in the organ culture medium, the epithelial cells undergo proliferation. They grow down into the space occupied by the dermis. Although, for the most part, separated from the stroma by basement membrane, the basement membrane erodes in places and epithelial cells invade the dermis at these sites (Fligiel and Varani, 1993; Zeigler *et al*, 1996a). Invasion in this model is accompanied by up-regulation of MMP-1 (interstitial collagenase) and MMP-9 (92-kD gelatinase B), and is blocked by the inclusion of tissue inhibitor of metalloproteinases-2 (TIMP-2) in the culture medium along with the exogenous growth factor (Varani *et al*, 1995; Zeigler *et al*, 1996b).

Efforts to understand how MMP-9 and MMP-1 are regulated during invasion in this model have shown the following: MMP-9 is elaborated primarily in the epidermis (Varani *et al*, 1995; Zeigler *et al*, 1996a); is up-regulated as a direct response of the epidermal cells to the exogenous EGF (Zeigler *et al*, 1999); and depends on signalling through mitogen activated protein kinase (MAPK) pathways (Zeigler *et al*, 1999). Specifically, signalling through both the extracellular signal-related kinase (ERK) pathway and the jun-N-terminal kinase (JNK) pathway occurs. This leads to formation of active c-*fos* and c-*jun* and the combination of these elements to form the AP-1 transcription complex. Interference with ERK signalling using a chemical inhibitor or with JNK signalling through use of a dominant-interfering mutant reduces formation of the AP-1 complex and MMP-9 transcription. In contrast, MMP-1 is primarily a dermal fibroblast product. MMP-1 induction reflects a fibroblast response to factors elaborated within the epidermis rather than a response to the exogenously-added EGF. At least two different classes of epidermal-derived factors appear to be important in promoting dermal MMP-1 production – i.e., factors that act through the interleukin-1 (IL-1) receptor, and factors that bind to and activate the EGF receptor (Moon *et al*, 2001). Little is known about the intracellular signalling events that are induced in fibroblasts by epidermal keratinocytes to bring about MMP-1 elaboration. The present study addresses this issue.

## MATERIALS AND METHODS

### Reagents

Human recombinant forms of EGF, heparin-binding EGF (HB-EGF), IL-1β and IL-1 receptor antagonist were obtained from R&D Systems (Minneapolis, MN, USA). U0126, a potent inhibitor of ERK1,2 kinase activity (Favata *et al*, 1998) and SB203580, an inhibitor of p38 kinase activity (Cuenda *et al*, 1995) were obtained from Calbiochem (San Diego, CA, USA). PD169540, a potent inhibitor of EGF receptor tyrosine kinase activity was a generous gift of Drs WL Leopold and David Fry of Pfizer Global Research and Development, Ann Arbor Laboratories, Ann Arbor, MI, USA. PD169540 is an acrylamide-substituted 4-anilinopyrido[d]pyrimidine designed to irreversibly alkylate Cys-773 within the ATP-binding pocket of c-erbB1 and c-erbB2 (EGF receptor family members) (Smaill *et al*, 2000).

### Human epidermal keratinocytes in monolayer culture

Normal human epidermal keratinocytes were obtained from foreskin tissue (obtained at circumcision) as described previously (Varani *et al*, 1994). They were maintained in monolayer culture using Keratinocyte Growth Medium (KGM) (Clonetics, Inc., Walkersville, MD, USA) as culture medium. KGM is a low-Ca^2+^ (0.15 mM) modification of MCDB-153 medium. It is supplemented with a mixture of growth factors including 0.1 ng ml^−1^ EGF, 0.5 μg ml^−1^ insulin, and 2% bovine pituitary extract. Growth was at 37°C in an atmosphere of 95% air and 5% CO_2_. Cells were sub-cultured by exposure to trypsin/EDTA and used at passage 2–3. In some experiments, the HaCaT line of immortalised human epidermal keratinocytes (Boukamp *et al*, 1988) was used in place of normal keratinocytes. HaCaT cells were propagated in exactly the same manner as low-passage keratinocytes, and used interchangeably with keratinocytes.

Culture fluid was prepared from keratinocyte or HaCaT cultures as follows: The cells were plated at 4×10^4^ cells per cm^2^ of surface area in 25-cm^2^ or 75-cm^2^ flasks. KGM was used as culture medium. When the cultures were approximately 75% confluent, the cells were washed twice in keratinocyte basal medium (KBM) and incubated for 72 h in KBM supplemented with 1.4 mM Ca^2+^. KBM consists of the same basal formulation as KGM but does not contain exogenous growth factors (EGF, insulin and pituitary extract). At the end of the incubation period, the culture fluid was collected and separated from cells and debris by low-speed centrifugation. The keratinocyte culture fluid obtained in this manner was used as a source of stimulating factor(s) for dermal fibroblast MMP-1 production.

### Human dermal fibroblasts in monolayer culture

Normal human dermal fibroblasts were isolated from neonatal foreskin as described previously (Varani *et al*, 1994). Fibroblasts were grown in monolayer culture using Dulbecco's Modified Minimal Essential Medium supplemented with non-essential amino acids and 10% foetal bovine serum (DMEM-FBS) as culture medium. Fibroblasts were maintained at 37°C in an atmosphere of 95% air and 5% CO_2_. Cells were sub-cultured by exposure to trypsin/EDTA and used at passage 2–3.

For assessment of MMP-1 production and for proliferation, dermal fibroblasts were plated at 2×10^4^ cells per cm^2^ of surface area in wells of a 24-well dish using DMEM-FBS as growth medium. After allowing cells to attach, the medium was removed and the cells washed twice in Ca^2+^-supplemented KBM. The cells were then incubated for 48 h in Ca^2+^-supplemented KBM alone or in a 50 : 50 (v v^−1^) mixture of Ca^2+^ -supplemented KBM and keratinocyte-conditioned medium. Additional reagents were added as indicated in Results. At the end of the incubation period, the cells were harvested by trypsinisation and enumerated using an automated particle counter. Culture fluid was clarified by low-speed centrifugation, following which it was assayed for MMP-1 as indicated below.

For assessment of signalling intermediates, cell extracts were prepared by lysis of the fibroblasts in buffer consisting of 20 mM Tris-HCl (pH 7.4), 2 mM sodium vanadate, 2 mM 10_4_ phosphate substrate, 100 mM NaCl, 1% NP40 detergent, 0.5% sodium deoxycholate detergent, 25 mg ml^−1^ each aprotinin, leupeptin and pepstatin and 2 mM each EDTA and EGTA. Lysis was performed at 4°C by scraping cells into the detergent-containing buffer and then fragmenting cell debris by several passes through a 26-gauge needle. Cell lysates were cleared by microcentrifugation at 12 000 G followed by incubation at 4°C for 25 min. The supernatant fluid was recovered and protein concentration of the lysate measured using the BioRad protein assay reagent (BioRad, Hercules, CA, USA).

### Substrate-embedded enzymography

SDS–PAGE substrate-embedded enzymography (zymography) was used to identify enzymes with collagenase and gelatinase activities. Assays were carried out exactly as described in a previous report (Gibbs *et al*, 1999). Briefly, denatured but non-reduced culture fluid samples were resolved in 7.5% SDS–PAGE gels prepared with the added incorporation of gelatin (1 mg ml^−1^) or β-casein (1 mg ml^−1^) prior to casting. After electrophoresis, gels were washed twice for 15 min in 50 mM Tris buffer containing 1 mM Ca^2+^, 0.5 mM Zn^2+^ and 2.5% Triton X-100. The gels were then incubated overnight in Tris buffer with 1% Triton X-100 and stained the following day with Coumassie Brilliant Blue 250-R. Following destaining, zones of enzyme activity were detected as regions of negative staining against the dark background. The zymograms were converted to negative images and digitised. Quantification was accomplished by determining the number of pixels in the negative images. Volumes of 5–35 μl of undiluted culture fluid were normally used for these assays; zones of activity were proportional to the quantity of culture fluid used. Gelatin zymography is useful for detection of MMP-2 (72-kD gelatinase A) in fibroblast cell culture fluids. β-casein zymography is useful for detection of MMP-1, which appears as a doublet in the 54-kD region of the gel. The β-casein zymographic bands co-migrated with purified MMP-1 as detected in Western blotting (Varani *et al*, 2000). Digestion of native, fibrillar type I collagen was observed in parallel with expression of MMP-1, and blocked in the presence of TIMP-2 or EDTA but not with a battery of serine proteinase inhibitors (Varani *et al*, 2000).

### Western blot analysis of total and phosphorylated forms of ERK and p38

Rabbit IgG antibodies to total and phosphorylated forms of ERK 1,2 and p38 were obtained from Cell Signaling (Beverly, MA, USA). Antibodies to total and phosphorylated forms of c-*jun* were obtained from Signal Transduction Laboratories (Lexington, KY, USA). Equivalent amounts of fibroblast extract (40 μg lane^−1^) were resolved by SDS–PAGE (12%) and transferred to nitrocellulose membranes by electroblotting using a BioRad mini transfer blotting apparatus. Membranes were then blocked in Ca^2+^- and Mg^2+^-free Tris-buffered saline (TBS) containing 5% Blotto. Membranes were then treated with antibodies to total or phosphorylated forms of the signalling intermediates (1 : 400–1 : 1000 dilutions) in TBS containing 0.5% Blotto and 0.1% Tween for 1 h. Following several rinses in TBS with 0.1% Tween, the membrane-bound antibodies were reacted with horseradish peroxidase-conjugated goat anti-rabbit antibody at 1 : 2000 dilution for 1 h. Protein-antibody complexes were detected by enhanced chemiluminescence (Cell Signaling) and visualised on light-sensitive autoradiographic film (Amersham, Pharmacia, London, UK). Digitisation and quantitation was performed as with zymography except that negative images were not constructed.

## RESULTS

### Epidermal keratinocyte stimulation of fibroblast MMP-1 elaboration and proliferation: Effects of ERK and p38 inhibitors

**Figure 1:**
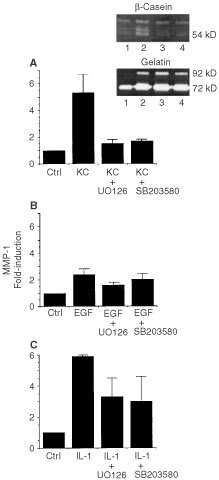
Fibroblast production of MMP-1 in response to stimulation by (**A**) keratinocyte-conditioned medium, (**B**) EGF and (**C**) IL-1β. Fibroblasts were exposed to culture medium alone or to a 50 : 50 mixture of culture medium and keratinocyte-conditioned medium. U0126 (10 μM) or SB203580 (15 μM) was included as indicated. At the end of the 2-day incubation period, MMP-1 was assessed by β-casein zymography. Quantitation was accomplished by densitometry scanning as described in the Materials and Methods section. Values shown are means and standard deviations based on four separate experiments. The insert shows a β-casein zymogram from one experiment. Lane 1 demonstrates MMP-1 levels (54 kD) in control fibroblast culture fluid; Lane 2 shows increased enzyme produced by fibroblasts exposed to keratinocyte-conditioned medium. Lanes 3 and 4 show the effects of U0126 and SB203580, respectively, on up-regulated enzyme production. A gelatin zymogram is shown for control. Neither inhibitor influenced expression of MMP-2 (72 kD).

Human dermal fibroblasts were exposed to 72-h keratinocyte-conditioned medium and incubated for 2 days. At the end of the incubation period, MMP-1 production and proliferation were assessed. Consistent with our recent findings (Moon *et al*, 2001), there was a greater than five-fold stimulation of MMP-1 production and an approximately two-fold increase in growth. Effects of two MAPK inhibitors – i.e., U0126 and SB203580 – on MMP-1 elaboration and growth were assessed. In the presence of 10 μM U0126, MMP-1 production was reduced by 88% and fibroblast growth was reduced by 95% (Figures 1A

and 2

**Figure 2:**
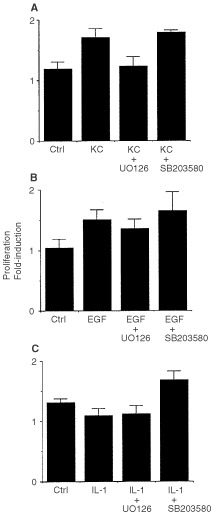
Fibroblast proliferation in response to stimulation by (**A**) keratinocyte-conditioned medium, (**B**) EGF and (**C**) IL-1β. Fibroblasts were exposed to culture medium alone or to a 50 : 50 mixture of culture medium and keratinocyte-conditioned medium. U0126 (10 μM) or SB203580 (15 μM) was included as indicated. At the end of the 2-day incubation period, cell number was assessed as described in the Materials and Methods section. Values shown are means and standard deviations based on four separate experiments, each with duplicate or triplicate samples.

A). The effects of U0126 on both biological responses were dose responsive. Activity (albeit reduced) was observed at a concentration of 1 μM. SB203580 also effectively blocked MMP-1 elaboration induced by keratinocyte-conditioned medium (84% inhibition at 15 μM), but this agent had no effect on the fibroblast proliferative response (Figure 2A). The fibroblast response to SB203580 was also dose-responsive, with activity seen at 1.5 μM. In contrast to the results with MMP-1, there was virtually no change in the level of MMP-2 in response to keratinocyte-conditioned medium and no effect with either U0126 or SB203580 (Figure 1, insert).

In a recent study it was demonstrated that ligand(s) which act through the EGF receptor and ligand(s) which act through the IL-1 receptor could together account for virtually all of the MMP-1 – inducing activity in keratinocyte culture fluid while EGF receptor agonists by themselves accounted for proliferation-inducing activity (Moon *et al*, 2001). We therefore utilised recombinant ligands for these receptors for comparative purposes. As shown in Figures 1B and 2B, EGF (10 ng ml^−1^) induced both MMP-1 elaboration and proliferation in fibroblasts. In comparison to keratinocyte culture fluid, MMP-1 – inducing activity of EGF was low (2.3-fold increase). Proliferation-inducing activity of purified recombinant EGF was comparable to that of keratinocyte culture fluid. Figures 1B and 2B also demonstrate that in the presence of U0126 (10 μM), EGF-induced MMP-1 production was reduced by 54% while EGF-induced proliferation was reduced by 34%. SB203589 (15 μM) had minimal inhibitory effect on MMP-1 production, and actually enhanced proliferation in response to EGF (Figures 1B and 2B).

Figures 1C and 2C show results from studies in which IL-1β (1 ng ml^−1^) was used to stimulate fibroblast production of MMP-1 and fibroblast proliferation. In the presence of IL-1β, there was a substantial induction of MMP-1 (5.3-fold), comparable to that seen in the presence of keratinocyte-conditioned medium. In contrast, IL-1β failed completely to stimulate fibroblast proliferation. In the presence of U0126, IL-1β – stimulated MMP-1 production was inhibited by 49%. In the presence of SB203580, 66% inhibition was observed. When proliferation rather than MMP-1 production was assessed, the presence of U0126 had no effect on the (lack of) response to IL-1β. Treatment with SB203580 along with IL-1β, however, actually enhanced proliferation slightly.

### ERK 1,2 phosphorylation in fibroblasts stimulated by 72-h keratinocyte-conditioned medium: comparison with EGF- and IL-1*β* -induced phosphorylation

Figure 3

**Figure 3:**
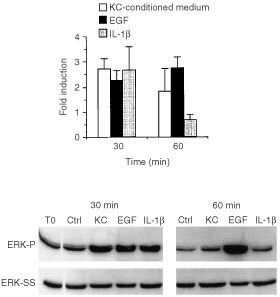
ERK 1,2 phosphorylation in fibroblasts in response to stimulation by keratinocyte-conditioned medium, EGF and IL-1β. Fibroblasts were exposed for 30 or 60 min to culture medium alone or to a 50 : 50 mixture of culture medium and keratinocyte-conditioned medium. At the end of the incubation period, extracts were prepared and assayed for phospho-ERK expression as described in the Materials and Methods section. Values shown are expressed as fold-induction relative to the level detected in cells exposed to culture medium alone. Values are means and standard deviations based on *n*=6 separate experiments for keratinocyte-conditioned medium and *n*=5 for EGF and IL-1β. The insert demonstrates a Western blot from one experiment. To: ERK 1,2 phosphorylation in an extract from cells exposed to culture medium alone at time-zero; Ctrl: ERK 1,2 phosphorylation in extracts from cells exposed to control medium alone at each time-point. ERK-P=phosphorylated ERK 1,2. ERK-SS=total ERK 1,2 protein.

demonstrates changes in ERK 1,2 phosphorylation at 30 and 60 min in fibroblasts exposed to conditioned medium from 72-h keratinocyte cultures. Increased phosphorylation was observed as early as 10 min after stimulation (earliest time-point examined; not shown), reached a maximum at 30 min and decreased to approximately 50% of the maximum value by 60 min. Changes in ERK 1,2 phosphorylation induced by EGF and IL-1β are shown for comparison. In the presence of EGF, ERK 1,2 phosphorylation was increased at the 30-min time-point and remained elevated through 60 min (longest time-point examined) (Figure 3). Although EGF was used as the EGF receptor agonist in most experiments, we also confirmed that a similar profile of ERK 1,2 phosphorylation occurred in the presence of HB-EGF. That is, at 10 ng ml^−1^, stimulation was seen at 30-min and remained elevated (approximately 50% of maximum) through at least 60 min (data not shown). IL-1β also induced ERK 1,2 phosphorylation. Enhanced phosphorylation was observed at 30 min. However, by 60 min, the level of phosphorylated ERK 1,2 had decreased to below baseline values. Under none of the conditions examined was there a measurable change in steady-state (total) ERK 1,2 protein (Figure 3).

The inhibitor U0126 was used in an effort to block ERK 1,2 phosphorylation. At a concentration of 10 μM, U0126 suppressed ERK 1,2 phosphorylation induced by keratinocyte-conditioned medium to below baseline levels (Table 1

**Table 1:**
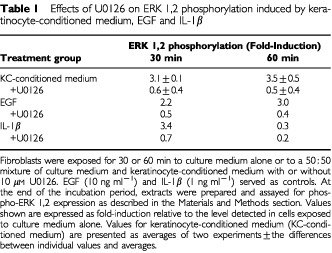
Effects of U0126 on ERK 1,2 phosphorylation induced by keratinocyte-conditioned medium, EGF and IL-1β

). Substantial inhibition was also observed when either EGF or IL-1β was used to induce ERK phosphorylation (Table 1).

### p38 phosphorylation in fibroblasts stimulated by 72-h keratinocyte-conditioned medium: comparison with phosphorylation induced by EGF- and IL-1β

Figure 4

**Figure 4:**
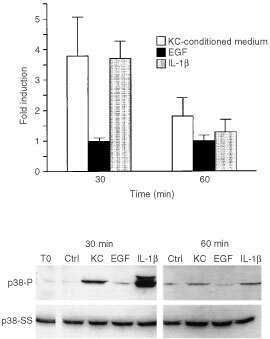
p38 phosphorylation in fibroblasts in response to stimulation by keratinocyte-conditioned medium, EGF and IL-1β. Fibroblasts were exposed for 30 or 60 min to culture medium alone or to a 50 : 50 mixture of culture medium and keratinocyte-conditioned medium. At the end of the incubation period, extracts were prepared and assayed for phospho-p38 expression as described in the Materials and Methods section. Values shown are expressed as fold-induction relative to the level detected in cells exposed to culture medium alone. Values are means and standard deviations based on *n*=6 separate experiments for keratinocyte-conditioned medium and *n*=5 for EGF and IL-1β. The insert demonstrates a Western blot from one experiment. To: p38 phosphorylation in an extract from cells exposed to culture medium alone at time-zero; Ctrl: p38 phosphorylation in extracts from fibroblast exposed to culture medium alone at each time-point. p38-P=phosphorylated p38. p38-SS=total p38 protein.

demonstrates p38 phosphorylation in fibroblasts under the same conditions as assessed with ERK 1,2. In the presence of 72-h keratinocyte culture fluid, p38 phosphorylation was strongly induced at 30 min. However, stimulation was transient. By 60 min, phosphorylation of p38 was decreased by 71% from the value seen at 30 min. When EGF was examined, p38 phosphorylation was not induced at either 30 or 60 min. In contrast, when IL-1β was used as the stimulus, the p38 phosphorylation pattern resembled that induced by keratinocyte culture fluid. Phosphorylation was induced at 30 min but had decreased to almost baseline level by 60 min. Under none of the conditions examined was there a measurable change in steady-state p38 protein (Figure 4).

**Figure 5:**
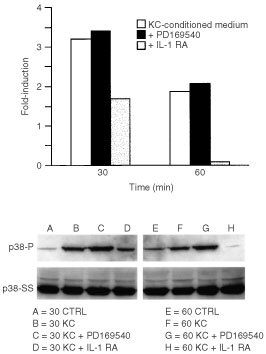
Effects of an IL-1 receptor antagonist and an EGF receptor tyrosine kinase antagonist (PD169540) on p38 phosphorylation induced by keratinocyte-conditioned medium. Fibroblasts were exposed for 30 or 60 min to culture medium alone or to a 50 : 50 mixture of culture medium and keratinocyte-conditioned medium with or without 50 μg ml^−1^ IL-1 receptor antagonist or 1 μM PD169540. At the end of the incubation period, extracts were prepared and assayed for phospho-p38 expression as described in the Materials and Methods section. Values shown are expressed as fold-induction relative to the level detected in cells exposed to culture medium alone. The insert demonstrates a Western blot from one experiment. p38-P=phosphorylated p38. p38-SS=total p38 protein.

Additional experiments were conducted in which an IL-1 receptor antagonist and a potent EGF receptor tyrosine kinase antagonist (PD169540) were examined for ability to modulate p38 phosphorylation induced by keratinocyte culture fluid. In the presence of IL-1 receptor antagonist (50 μg ml^−1^), p38 phosphorylation was inhibited by 80% at 30 min and virtually 100% at 60 min. Not surprising in light of the failure of EGF to induce p38 phosphorylation, PD169540 (1 μM) was completely ineffective in preventing p38 phosphorylation (Figure 5

).

**Figure 6:**
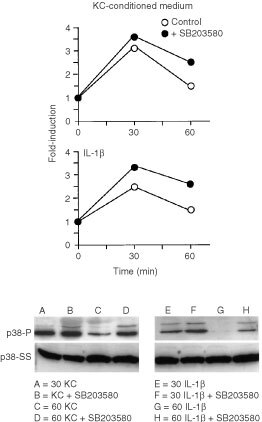
Effects of SB203580 on p38 phosphorylation induced by keratinocyte-conditioned medium or IL-1β. Fibroblasts were exposed for 30 or 60 min to culture medium alone or to a 50 : 50 mixture of culture medium and keratinocyte-conditioned medium with or without 15 μM SB203580. At the end of the incubation period, extracts were prepared and assayed for phospho-p38 expression as described in the Materials and Methods section. Values shown are expressed as fold-induction relative to the level detected in cells exposed to culture medium alone. The insert demonstrates a Western blot from one experiment. p38-P=phosphorylated p38. p38-SS=total p38 protein.

In the next set of experiments, effects of SB203580 on p38 phosphorylation induced by keratinocyte culture fluid was assessed. Consistent with the findings presented in Figure 4, p38 phosphorylation was increased at 30 min under control conditions (i.e., in the presence of keratinocyte-conditioned medium) and then decreased almost to baseline by 60 min. In the presence of both the keratinocyte culture fluid and the inhibitor, the level of phosphorylation seen at 30 min was increased slightly over that seen with culture fluid alone. More impressively, the decrease seen at 60 min in the presence of culture fluid was substantially inhibited in the presence of SB203580 (Figure 6

). Similar results were observed when IL-1β was used as the stimulus in place of keratinocyte culture fluid (Figure 6).

**Figure 7:**
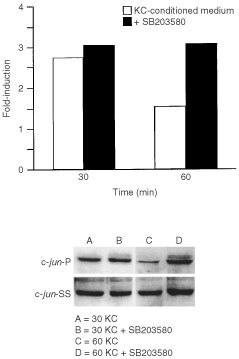
c-*jun* phosphorylation in fibroblasts exposed to keratinocyte-conditioned medium in the presence or absence of SB203580. Fibroblasts were exposed for 30 or 60 min to culture medium alone or to a 50 : 50 mixture of culture medium and keratinocyte-conditioned medium with or without 15 μM SB203580. At the end of the incubation period, extracts were prepared and assayed for phospho-c-*jun* expression as described in the Materials and Methods section. Values shown are expressed as fold-induction relative to the level detected in cells exposed to culture medium alone. The insert demonstrates a Western blot from one experiment. c-*jun*-P=phosphorylated c-*jun*. c-*jun*-SS=total c-*jun* protein.

Finally, keratinocyte culture fluid was examined for ability to stimulate c-*jun* phosphorylation in the absence and presence of SB203580. In the absence of the inhibitor, there was an approximately 3.6-fold increase in phosphorylation at 30 min, which decreased to 1.5-fold at 60 min. The presence of SB203580 had virtually no effect at 30 min, but completely inhibited the decrease at 60 min (Figure 7

). The level of c-*jun* steady-state protein remained constant (Figure 7).

## DISCUSSION

MMPs are thought to play a major role in tissue destruction associated with tumour invasion (reviewed in: Stetler-Stevenson *et al*, 1993; Stamenkovic, 2000; Herouy, 2001). Utilising an organ culture model of epithelial cell invasion in skin, we have demonstrated that stromal invasion in this model was accompanied by the up-regulation of MMP-9 and MMP-1 and inhibited in the presence of exogenous TIMP-2 (Varani *et al*, 1995; Zeigler *et al*, 1996b). MMP-9 was elaborated in the epidermis as a direct response to the exogenous growth factors used to stimulate invasion. In contrast, MMP-1 was produced largely in the stroma. Induction of this enzyme occurred as a response to factors elaborated by the epidermis. At least two distinct epithelial cell factors – one, a ligand for the EGF receptor, and the other an agonist for the IL-1 receptor – appeared to be responsible for enzyme induction (Moon *et al*, 2001). In the present study we have examined MAPK signalling events that underlie MMP-1 induction in fibroblasts following exposure to conditioned medium from keratinocyte cultures. To summarise, activation of both ERK and p38 pathways occurs in fibroblasts exposed to keratinocyte-conditioned medium, and both pathways contribute to enzyme induction. Several important issues can be addressed relative to these findings.

One is the relationship between MAPK signalling events triggered by keratinocyte-conditioned medium and MMP-1 induction by the same factors. Our data indicate that activation of ERK 1,2 by keratinocyte-conditioned medium reflects the summation of action of ligands for both EGF receptor and IL-1 receptor, while p38 activation is mediated primarily (if not exclusively) by IL-1 receptor agonists. This is based on the similarity of phosphorylation patterns seen at 30 and 60 min after exposure of the cells to either keratinocyte-conditioned medium or to prototype ligands for the two receptors (i.e., EGF and IL-1β) as indicated in Figures 3 and 4. Consistent with this, the ERK pathway inhibitor U0126 strongly inhibited MMP-1 production in fibroblasts exposed to keratinocyte culture medium, and concomitantly reduced MMP-1 production in fibroblasts exposed to either EGF or IL-1β. The p38 pathway inhibitor SB203580 also inhibited MMP-1 induction by keratinocyte-conditioned medium. This inhibitor blocked MMP-1 production in response to IL-1β, but in contrast to U0126, had no effect of MMP-1 production following EGF stimulation. Taken together, these data indicate that EGF-like ligands induce MMP-1 production in fibroblasts by acting through the ERK pathway (independent of p38). In contrast, the IL-1 receptor agonist(s) appear to stimulate MMP-1 production by concomitantly signalling through both ERK and p38 pathways.

It should be noted that while the actual keratinocyte-derived ligands for the EGF and IL-1 receptors were not identified in this study, past work has shown that HB-EGF is a likely candidate for the EGF receptor while IL-1β is the major keratinocyte ligand for the IL-1 receptor. Keratinocytes produce several EGF receptor agonists, but of these, only HB-EGF is strongly up-regulated in organ-cultured skin (Stoll *et al*, 1997). Furthermore, it was shown in a recent study that epidermal hyperplasia in organ-cultured skin could be inhibited with an antibody to HB-EGF (Varani *et al*, 2001). IL-1β is likely to be the major agonist for the IL-1 receptor because although both IL-1α and IL-1β are elaborated by keratinocytes, the β form is the major isoform secreted into the culture medium while the IL-1α remains largely cell-associated (Maas-Szabowski *et al*, 1999; Maas-Szabowski *et al*, 2000).

While ligands for both EGF and IL-1 receptors participate in the induction of MMP-1 elaboration, the data suggest that ligands acting through the IL-1 receptor provide the predominant stimulus. In the presence of EGF, a 2.3-fold induction of MMP-1 was seen while in the presence of IL-1β, induction was 5.5-fold. Both EGF (as well as HB-EGF) and IL-1β were examined over a wide range of concentrations and these were the maximal values obtained. More importantly, it was demonstrated in a recent study that a potent EGF receptor tyrosine kinase inhibitor completely blocked MMP-1 induction by EGF but was only modestly effective in blocking stimulation by keratinocyte-conditioned medium. In contrast, IL-1 receptor antagonist was highly-effective in blocking MMP-1 induction by either IL-1β or keratinocyte culture fluid (Moon *et al*, 2001).

A corollary issue is the relative contribution of each of the two signalling pathways to MMP-1 induction and the possible interaction between the two. Greatly reduced MMP-1 production in the presence of either the ERK pathway inhibitor or the p38 pathway inhibitor strongly suggests that signalling through both MAPK pathways is required for induction of MMP-1 by keratinocyte-conditioned medium. The contribution of EGF (acting through the ERK pathway) appears to be independent of p38, based on the lack of effect of the p38 inhibitor in preventing EGF-induced MMP-1 up-regulation. The fact that c-*jun* is still phosphorylated (and presumably activated) in the presence of SB203580, provides a p38-independent path to the formation of the AP-1 transcription complex (Zeigler *et al*, 1999). This does not appear to be the case with the component of stimulation due to IL-1β. Stimulation of MMP-1 production via IL-1 receptor activation can be blocked by inhibiting either ERK or p38 signalling. Since almost identical results were achieved when either keratinocyte-conditioned medium or recombinant IL-1β was used as agonist, the presumption is IL-1 receptor activation is the major pathway leading to MMP-1 up-regulation and that signalling through both ERK and p38 pathways is required for maximal stimulation via this receptor.

The findings presented here are of interest in regard to what has been seen in other cell types or in fibroblasts stimulated with other agonists. Recent studies have shown that in human dermal fibroblasts, MMP-1 – inducing agents as diverse as okadaic acid, ceramide and EMMPRIN (extracellular matrix proteinase inducer) are susceptible to inhibition of either ERK or p38 signalling (Lim *et al*, 1998; Reunanen *et al*, 1998; Westermarck *et al*, 1998). In contrast, MMP-1 induced by basic fibroblast growth factor is susceptible to ERK inhibition but not to inhibition of p38 signalling (Brauchle *et al*, 2000). Taken together with our current findings, this suggests that ‘classical’ growth factors may provide a route to MMP-1 up-regulation not shared with many other agonists. Our own past studies with MMP-9 induction in keratinocytes is consistent with this. In those studies, it was observed that EGF-induced signalling occurred through ERK and JNK pathways without significant activation of p38 (Zeigler *et al*, 1999). In keratinocytes, MMP-9 production was blocked with an ERK pathway inhibitor (PD98059) or when a dominant interfering JNK mutant was introduced into the cells.

Another issue is the relationship between MMP-1 induction in fibroblasts exposed to keratinocyte-conditioned culture medium and the concomitant induction of fibroblast proliferation. Fibroblast proliferation was induced to a similar extent by keratinocyte-conditioned medium and exogenous EGF, but there was no stimulation by IL-1β. The ERK pathway inhibitor reduced cell growth due to either stimulus while the p38 pathway inhibitor was without effect. These data suggest that fibroblast proliferation in response to keratinocyte culture fluid reflects growth factor activation of the ERK pathway. This is consistent with previous findings in other cell types (Dimon-Gadal *et al*, 1998; Zeigler *et al*, 1999; Chung *et al*, 2000). How ERK promotes fibroblast proliferation is not fully understood. The proliferative response may reflect activation of the cell cycle progression factors such as cyclin D2 by ERK (Chung *et al*, 2000). Of interest, fibroblasts treated with the p38 inhibitor had a small but reproducible increase in growth regardless of whether the cells had been stimulated with either keratinocyte-conditioned medium or EGF. Even in the presence of IL-1β, where there was no proliferation in the absence of inhibitor (Proliferation index of 0.98), there was a slight positive response in the presence of SB203580 (Proliferation index of 1.26). How this is brought about is not known. Perhaps signalling through p38 results in the production of growth-inhibiting molecules. Alternatively, signalling through p38 may simply provide more substrate for interaction with down-stream elements of ERK, which may, in turn, utilise activated ERK and prevent it actions elsewhere. Additional experiments will be needed to fully address this and other possibilities.

In summary, while no *in vitro* culture system can fully mimic what occurs *in vivo*, these data allow one to suggest that keratinocyte-fibroblast interactions are mediated by multiple stimulating agents acting on specific receptors to induce signalling through different MAPK pathways in order to jointly alter expression of key biological functions.
